# A novel polymorphism in the fatty acid desaturase 2 gene (*Fads2*): A possible role in the basal metabolic rate

**DOI:** 10.1371/journal.pone.0213138

**Published:** 2019-02-28

**Authors:** Magdalena Czajkowska, Paweł Brzęk, Paweł Dobrzyń

**Affiliations:** 1 Institute of Biology, University of Białystok, Białystok, Poland; 2 Laboratory of Molecular Medical Biochemistry, Nencki Institute of Experimental Biology, Polish Academy of Sciences, Warsaw, Poland; Universite du Quebec a Montreal, CANADA

## Abstract

Fatty acyl composition of cell membrane lipids, particularly the abundance of highly unsaturated docosahexaenoic fatty acid (22:6n-3, DHA), is likely to be an important predictor of basal metabolic rate (BMR). Our study was performed using two lines of laboratory mice divergently selected for either high or low BMR. We describe a novel single nucleotide polymorphism in the *Fads2* gene encoding Δ6-desaturase, a key enzyme in the metabolic pathways of polyunsaturated fatty acids (PUFAs). The allele frequencies of *Fads2* were significantly different in both lines of mice. The analysis of genetic distances revealed that the genetic differentiation between the two studied lines developed significantly faster at the *Fads2* locus than it did at neutral loci. Such a pattern suggests that the *Fads2* polymorphism is related to the variation in BMR, i.e. the direct target of selection. The *Fads2* polymorphism significantly affected abundance of several PUFAs; however, the differences in PUFA composition between lines were compatible with the difference in frequency of *Fads2* alleles only for DHA. We hypothesize that the polymorphism in the *Fads2* gene affects the BMR through modification of DHA abundance in cell membranes. This may be the first example of a significant link between a polymorphism in a gene responsible for fatty acyl composition and variation in BMR.

## Introduction

Intraspecific variation in the basal metabolic rate (BMR) plays a profound role in both evolution and medicine [1–3]. However, little is understood about its molecular and genetic background [3]. Although BMR is a complex and polygenic trait [3], its intraindividual variation can sometimes be significantly modified by polymorphisms in a single gene [4].

The ‘membrane pacemaker theory of metabolism’ (MPTM) assumes that the fatty acyl composition of cell membrane lipids can modulate BMR by affecting the biological properties of membranes [5, 6]. Polyunsaturated fatty acids (PUFAs), especially docosahexaenoic acid (22:6n-3, DHA), are hypothesized to be particularly important predictors of cell membrane properties and BMR [6]. Although there is a considerable level of interest in studying MPTM (reviewed in [7]), only a few studies have directly tested the significance of the essential mechanisms underlying the postulated links between cell membrane fatty acyl composition and BMR [7]. Biochemical pathways of fatty acid synthesis are well known [8], and polymorphism of genes encoding enzymes involved in these pathways can significantly affect different physiological parameters [9–11]. However, to the best of our knowledge, polymorphism of genes involved in PUFA synthesis has not yet been shown to be related to individual variation in BMR.

Subjects in our experiment were laboratory mice that were selectively bred into high (H-BMR) or low (L-BMR) levels of BMR [12]. Although neither line is replicated, differences in the BMR between these lines are large enough to assert that they represent a genuine effect of selection rather than genetic drift [12, 13]. Because the relative differences in BMR between the lines exceeds 50%, these lines offer a unique model for investigating mechanisms beyond intraspecific variation in BMR. Although between-line differences in BMR mainly reflect different sizes of internal organs, an earlier study revealed that selection also significantly affected fatty acyl composition of cell membranes [13]. This result confirmed the presence of a direct link between cell membrane composition and BMR. However, it may reflect molecular mechanisms other than those proposed by the MPTM hypothesis (there was more double bonds in L-BMR line, i.e. the direction of change in fatty acyl composition was opposite to that predicted by MPTM; [13]).

We genotyped mice from both selected lines at the *Fads1*, *Fads2*, *Elovl-2* and *Elovl-5* genes, which respectively encode Δ5-desaturase, Δ6-desaturase, ELOVL2 and ELOVL5 elongases. This is the complete set of enzymes involved in biosynthesis of n-6 and n-3 PUFAs from dietary precursors (respectively, linoleic acid, 18:2n-6, LA and linolenic acid, 18:3n-3, ALA) in mice [8]. We also quantified the abundance of several PUFAs that are either substrates or products of n-6 and n-3 metabolic pathways, including LA, ALA, arachidonic acid (20:4n-6, ARA), eicosapentaenoic acid (20:5n-3, EPA), and docosahexaenoic acid (DHA). We expected that any potential polymorphism in the studied genes would likely be affected by selection and would also be related to variation in BMR or PUFA composition.

## Materials and methods

### Animals

Subjects in our experiment were males from two lines of outbred Swiss Webster laboratory mice selectively bred towards low (L-BMR) or high (H-BMR) body mass-corrected BMR. Details of the selection procedures and maintenance conditions are described elsewhere [12]. Briefly, the BMR of 12–16-week-old mice was measured for 3 h in an open-circuit respirometry system at an ambient temperature of 32°C (i.e., within the thermoneutral zone of mice). Males and females characterized by the highest and lowest mass-corrected BMR were chosen as progenitors of the H-BMR and L-BMR selection lines, respectively. A similar procedure has been repeated in every subsequent offspring generation, yielding significant differentiation of the lines with respect to BMR without simultaneous changes in body mass. Although both lines came from an unreplicated selection experiment, differences in BMR between the lines are large enough to claim that they represent a genuine change in the frequency of alleles directly related to BMR rather than genetic drift [12, 13]. The rearing conditions applied during the selection experiment (ambient temperature of 23°C, 12:12 light-dark cycle, food and water offered *ad libitum*) were also applied for mice used in the present experiment.

We used mice from generation F22 (40 L-BMR and 38 H-BMR; these animals were subjects of the experiment described in [13]), and generation F32 (61 L-BMR and 59 H-BMR). Mice were sacrificed by cervical dislocation at 4–6 months of age. Livers and tail tips (only in F32) were preserved in liquid nitrogen and stored at -80°C. In F32, liver fragments from 16 mice were also preserved in the RNAlater stabilization reagent (Qiagen, Hilden, Germany) for RNA extraction. All procedures were approved by the Ethical Committee on Animal Experimentation in Białystok ‘Local Ethical Committee on Testing Animals, Medical University of Białystok, Poland’ (permits 2003/34, 4/2009, 5/2010).

### Genotyping and identification of polymorphic sites in the *Fads1*, *Fads2*, *Elovl-2* and *Elovl-5* genes

We isolated RNA from liver samples of 8 males from each of the selected lines (F32) to obtain coding regions of the studied genes. RNA was extracted and purified using the RNeasy Mini Kit (Qiagen, Hilden, Germany) from ~10 mg of the homogenized liver tissue samples after the DNAse treatment step. First-strand cDNA was synthesized using Omniscript Reverse Transcriptase (Qiagen, Hilden, Germany) in 20 μL reaction mixture containing 6 μL of template RNA, 2 μL of Oligo(dT)^18^ primer (0.5 μg/μL, Invitrogen), 1 μL of RNase inhibitor (10 U/μL), 1 μL of RT and 6 μL of RNase-free water. The reaction mixture was incubated at 37°C for 60 min.

Primers for all genes amplifications were designed using Primer3 (v 0.4.0) software [14]. The forward primer of each gene spans the untranslated region before exon 1, and the reverse primers were located in the untranslated region after the last exon. Therefore, we could obtain almost the entire sequence of each gene (see S1 Table). PCRs for each primer pair were carried out in 5 μL volumes, and the reaction mixtures consisted of 2 μL of cDNA (~20 ng), 1.7 μL of Qiagen Multiplex PCR Master Mix (1x), 0.3 μL of primer mixture (0.2 μM of each primer) and 1 μL of RNase-free water. The polymerase chain reaction cycling scheme was as follows: 15 min at 95°C followed by 37 (*Fads1*, *Elovl-5*), 30 (*Fads2*) or 35 (*Elovl-2*) cycles of 30 s at 94°C; 90 s at 60°C (*Fads1*), 58°C (*Fads2*, *Elovl-5*), or 57°C (*Elovl-2*); 60 s at 72°C and the final extension step of 30 min at 60°C. PCR products were purified with the Clean-Up kit (A&A Biotechnology) and sequenced in both directions with the BigDey Terminator v3.1 Cycle Sequencing Kit (Applied Biosystems). Sequencing reaction products were purified with the ExTerminator kit (A&A Biotechnology) and separated on a 3130 Genetic Analyzer (Applied Biosystems). The DNA sequences were aligned in BioEdit v 7.0.4.1 [15] and revised manually for polymorphic site detection.

We extracted total DNA from tail tips or livers of all the remaining animals using the Genomic Mini kit (A&A Biotechnology) to omit the RT reaction step. Due to the length of the intron sequences, we designed two new primer pairs (using gDNA instead of cDNA as template) for shorter sequences which included both previously identified polymorphic sites in the *Fads2* gene (S1 Table). PCRs were performed, and the products were sequenced as described above. Our sequences are available under the GenBank accession numbers HQ2264057–HQ2264060 and KF987079–KF987081.

The Consensus CDS (CCDS) database (http://www.ncbi.nlm.nih.gov/CCDS/CcdsBrowse.cgi) was used to identify protein coding regions for further determination of the type of observed mutations (synonymous/nonsynonymous) in the *Fads2* gene.

### Genetic background and detection of candidate loci for selection

We used microsatellite loci as a neutral genetic background for estimation of the force of genetic drift between the studied lines of mice as well as for outlier locus detection. The genotypes of all mice were characterized at 10 microsatellite loci (S2 Table), which were derived from the Mouse Genome Informatics (www.informatics.jax.org) resource. To identify outlier loci, we used the program LOSITAN [16], which is based on fdist method, as described previously [17]. Shortly, this method describes the expected distribution of values of Wright's inbreeding coefficient *F*_ST_ versus *H*e (expected heterozygosity) under an island model of migration with neutral markers. This distribution is used to identify outlier loci that have excessively high or low *F*_ST_ when compared to neutral alleles. Such outlier loci are candidates for being subject to selection (respectively, directional or balancing). This analysis was only carried out in F22 since the effect of selection was masked by the effects of genetic drift in F32 (see Results).

### Analysis of PUFA content in total liver fatty acids

This analysis was carried out only for F32. We analyzed fatty acyl composition in the liver because this organ plays a key role in metabolism of PUFAs [18, 19] and both desaturases show high expression in this organ [20, 21]. Moreover, since selection for high and low BMR significantly affected liver size [12, 13], we expected that selection likely also modified the biochemical properties of this organ. Total lipids were extracted from the livers according to the methods of Folch [22], and their fatty acyl composition was analyzed by gas-lipid chromatography as described elsewhere [23]. We expressed relative molar amounts of each PUFA (LA, ALA, ARA, EPA, and DHA) as their relative percentage of all fatty acyl chains.

### Statistical analyses

We used CERVUS 3.0.3 [24] to estimate allele frequencies in the *Fads2* gene and in the 10 studied microsatellite loci. Fisher’s exact test was used to compare frequencies of both *Fads2* alleles between the studied lines in each generation. The genotype frequency data were statistically tested for deviation from the Hardy-Weinberg proportions using Genepop 4.0 [25]. The genetic differentiation in the *Fads2* gene between the selected lines of mice in F22 and F32 were estimated by the calculation of *F*_ST_ values (which quantify the variance of allele frequencies between two populations [26]) using FSTAT 2.9.3 [27]. The significance of *F*_ST_ values was ascertained with 1,000 permutations and interpreted using Wright’s scale [28]. The average *F*_ST_ value was also estimated for 10 microsatellite loci, which as neutral markers enable control for the force of the genetic drift. The 95% confidence interval (CI) was estimated in FSTAT 2.9.3.

We analyzed the effect of selection on the BMR and PUFA content with ANOVA using the effect of the selected line as the main factor, the effect of the generation as a random factor, and the effect of body mass as a covariate (the last two effects were used only for analysis of BMR). Body mass, BMR, and abundance of ALA were log-transformed to improve homogeneity of variation. Since the frequency of the *Fads2* genotypes differed significantly between H-BMR and L-BMR lines (see Results), the effect of genotype is almost indistinguishable from the effect of line affiliation, and neither effect can be correctly parameterized by a single model. Therefore, we used two approaches to compare *Fads2* genotypes: (1) we pooled mice from both lines together, and analyzed the effect of the *Fads2* genotype on BMR and the content of PUFAs using ANOVA with the effect of genotype as the main factor, the effect of generation as a random factor, and the effect of body mass as a covariate (the last two effects were used only for the analysis of BMR); (2) we calculated (independently for each line and each generation) the mean value and standard deviation of each analyzed trait. We then calculated standard scores (again, independently for each line and each generation) by subtracting the mean value from an individual observation and dividing the result by the standard deviation. We then pooled standard scores from all lines/generations together and analyzed using ANOVA with the effect of genotype as the main factor and (only for BMR) the effect of body mass as a covariate. Thereby, we tested the impact of the *Fads2* genotype on intraline and intragenerational variation in PUFA content and BMR. We hypothesized that the effect of the *Fads2* genotype was presumably overestimated by the first approach and underestimated by the second approach.

We tested differences between the *Fads2* genotypes with Tukey post hoc tests. The analyses were carried out using the MIXED procedure in SAS 9.3 software.

## Results

### Polymorphism of the genes involved in the n-6 and n-3 PUFA metabolic pathways

We successfully amplified the entire translated region of the *Fads1*, *Fads2*, *Elovl-2* and *Elovl-5* genes. Only the *Fads2* gene showed two polymorphic sites in the 1,302-bp translated sequence (see S1 Fig). The first single nucleotide polymorphism (SNP), G/A, was identified at position 167 in exon 3 and position 487 in the gene. This substitution was nonsynonymous and resulted in two alleles of the *Fads2* gene which encoded two variants of the Δ6-desaturase enzyme differing in their amino acid identity at position 163 (valine: allele G, isoleucine: allele A). The second SNP, C/T, was located at position 88 in exon 9 and position 1,068 in the *Fads2* gene. However, this mutation was synonymous (phenylalanine; F/F).

The G/A polymorphism in the *Fads2* gene was found in both studied lines and in both generations (Table 1). Allele A was significantly more abundant in the L-BMR line than in the H-BMR line (two-tailed Fisher’s exact test, *P* < 0.001 for both generations; Table 1). The Hardy-Weinberg equilibrium at the *Fads2* gene was confirmed in both selected lines of both generations (*P* > 0.05). Pairwise comparisons of both allele and genotype frequencies, based upon *F*_ST_ values at the *Fads2* locus, suggest very great (in Wright’s scale) and significant genetic differentiation between the selected lines in F22 (L-BMR v H-BMR; *F*_ST_ = 0.273, permutation testing, *P* < 0.001) and moderate genetic differentiation in F32 (L-BMR v H-BMR; *F*_ST_ = 0.140, *P* < 0.001).

**Table 1:** Number of mice with *Fads2* genotypes and frequencies of *Fads2* alleles.

	F22		F32	
	L-BMR	H-BMR	L-BMR	H-BMR
Number of mice with *Fads2* genotypes				
AA	4	0	6	2
AG	25	5	29	8
GG	11	33	26	49
Frequencies of *Fads2* alleles				
*F*_A_	0.41	0.07	0.34	0.10
*F*_G_	0.59	0.93	0.66	0.90

### Genetic drift between the studied lines of mice and outlier loci detection

The genotypes obtained for the 10 neutral microsatellite loci were used to calculate the average *F*_ST_ values between the lines of selected mice from F22 and F32 to measure the force of genetic drift. The corresponding *F*_ST_ estimates between the H-BMR and L-BMR lines of mice from F22 indicated a moderate and significant level of genetic differentiation (*F*_ST_ = 0.096, 95% CI: 0.029–0.171, *P* < 0.001), whereas 10 generations later (F32), the *F*_ST_ value increased to 0.224 (95% CI: 0.111–0.314, *P* < 0.001).

**Fig 1:**
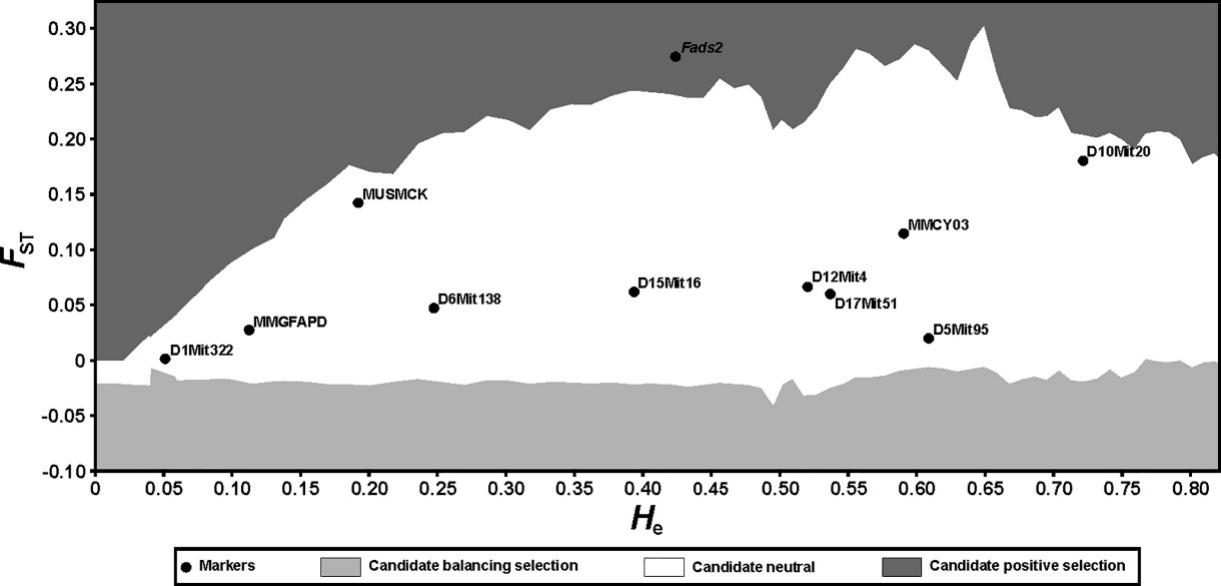
Identification of candidate loci influenced by selection for basal metabolic rate. Comparison of *F*_ST_ and the expected heterozygosity (*H*_e_) values at polymorphic loci (10 microsatellites and the *Fads2* gene). The analysis was carried out using LOSITAN software. All microsatellite loci were found to be candidate neutral markers (located in the white region of the figure), whereas the *Fads2* gene had excessively high *F*_ST_ compared to neutral expectation and was found to be candidate positive selection (the dark grey region).

Values of *F*_ST_ for neutral loci increased between F22 and F32, suggesting that the effect of genetic drift increased over time. In fact, the *F*_ST_ for *Fads2* in F32 was within the 95% CI for neutral loci, indicating that the potential effect of selection at this locus was undistinguishable from the effect of genetic drift. Therefore, we carried out an outlier locus detection test for F22 only. Using the genotypic data obtained from the 10 neutral microsatellite loci and the corresponding *F*_ST_ value at the *Fads2* gene between the H-BMR and L-BMR lines, we could distinguish the effects of genetic drift and selection. Analysis conducted using the LOSITAN program revealed that the genetic differentiation among lines was significantly greater at the *Fads2* locus than at any of the 10 microsatellite loci, indicating that this locus may be under selection (Fig 1).

### Effects of selection and polymorphism in *Fads2* on BMR

**Fig 2:**
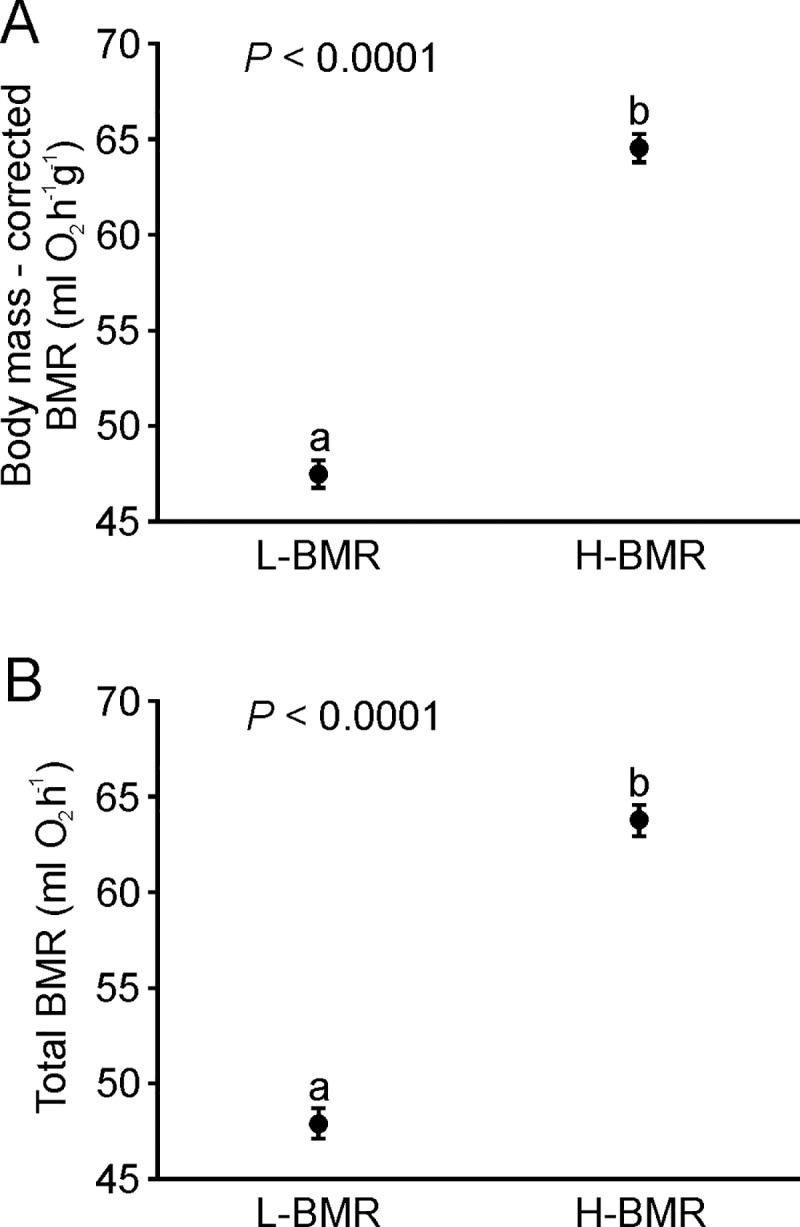
Effect of selection on BMR. Effect of selection on BMR with (A), and without (B) correction for body mass. Data are presented as LS means (A) and means (B) ± s.e.m. Different letters indicate significant differences between lines. Sample size was *n* = 100 for line L-BMR and *n* = 97 for line H-BMR.

H-BMR mice had higher BMR than did L-BMR mice (effect of selection: F_1, 192_ = 308, *P* < 0.0001, effect of generation: F_1, 192_ = 6.44, *P* = 0.012, interaction between the effect of selection and effect of generation: F_1, 192_ = 31.4, *P* < 0.0001, effect of body mass: F_1, 192_ = 80.9, *P* < 0.0001; Fig 2A). The significant interaction between the effect of selection and the effect of generation revealed continued selection between F22 and F32; however, the difference in BMR between the H-BMR and L-BMR lines was highly significant (*P* < 0.0001) in both generations. The effect of selection was still highly significant when BMR was not corrected for body mass (effect of selection: F_1, 193_ = 215, *P* < 0.0001, effect of generation: F_1, 193_ = 0.66, *P* = 0.42, interaction between effect of selection and effect of generation: F_1, 193_ = 25.9, *P* < 0.0001; difference in BMR between lines H-BMR and L-BMR in each generation: *P* < 0.0001; Fig 2B).

**Fig 3:**
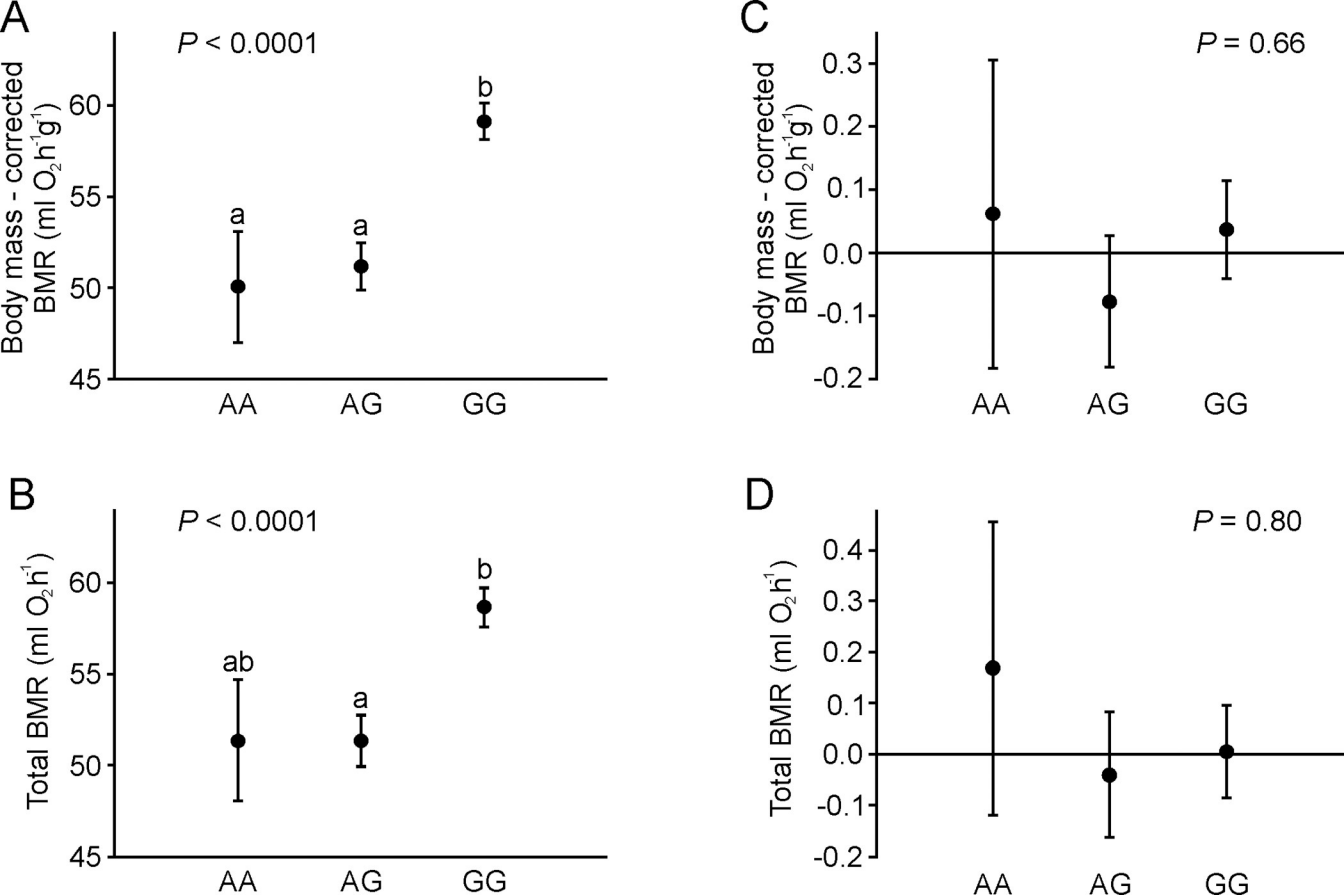
Effect of the *Fads2* genotype on BMR. The effect of the *Fads2* genotype on BMR with (A), and without (B) correction for body mass, and the effect of the *Fads2* genotype on standardized scores of BMR with (C) and without (D) correction for body mass. Data are presented as LS means (A, C) and means (B, D) ± s.e.m. Different letters indicate significant differences between genotypes. Sample size was *n* = 12 for AA genotype, *n* = 66 for AG genotype, and *n* = 119 for GG genotype.

When we analyzed mice from both lines together, the *Fads2* genotype had a significant effect on BMR (effect of genotype: F_2, 192_ = 16.2, *P* < 0.0001, effect of generation: F_1, 192_ = 3.96, *P* = 0.048, effect of body mass: F_1, 192_ = 37.6, *P* < 0.0001; Fig 3A). Mice with the GG genotype had higher BMR than did mice with AG (*P* < 0.0001) and AA genotypes (*P* = 0.01). BMR did not differ between mice possessing allele A (AA/AG genotypes; *P* = 0.97). Similar results were found when BMR was not corrected for body mass (effect of genotype: F_2, 193_ = 11.4, *P* < 0.0001, effect of generation: F_1, 193_ = 0.70, *P* = 0.4; Fig 3B). Mice with genotype GG had higher BMR than that of mice with the AG genotype (*P* < 0.0001) and tended to have a higher BMR than did mice with the AA genotype (*P* = 0.068). BMR did not differ between mice possessing allele A (AA/AG genotypes; *P* > 0.99). However, when calculations were carried out on standardized values, the *Fads2* genotype had no significant effect on standard scores of BMR (analysis with correction for body mass: effect of genotype: F_2,193_ = 0.42, *P* = 0.66, effect of standard scores of body mass: F_1,193_ = 76, *P* < 0.0001; Fig 3C; analysis without correction for body mass: F_2,194_ = 0.23, *P* = 0.80; Fig 3D).

### Effect of selection and polymorphism in the *Fads2* gene on PUFA composition

**Fig 4:**
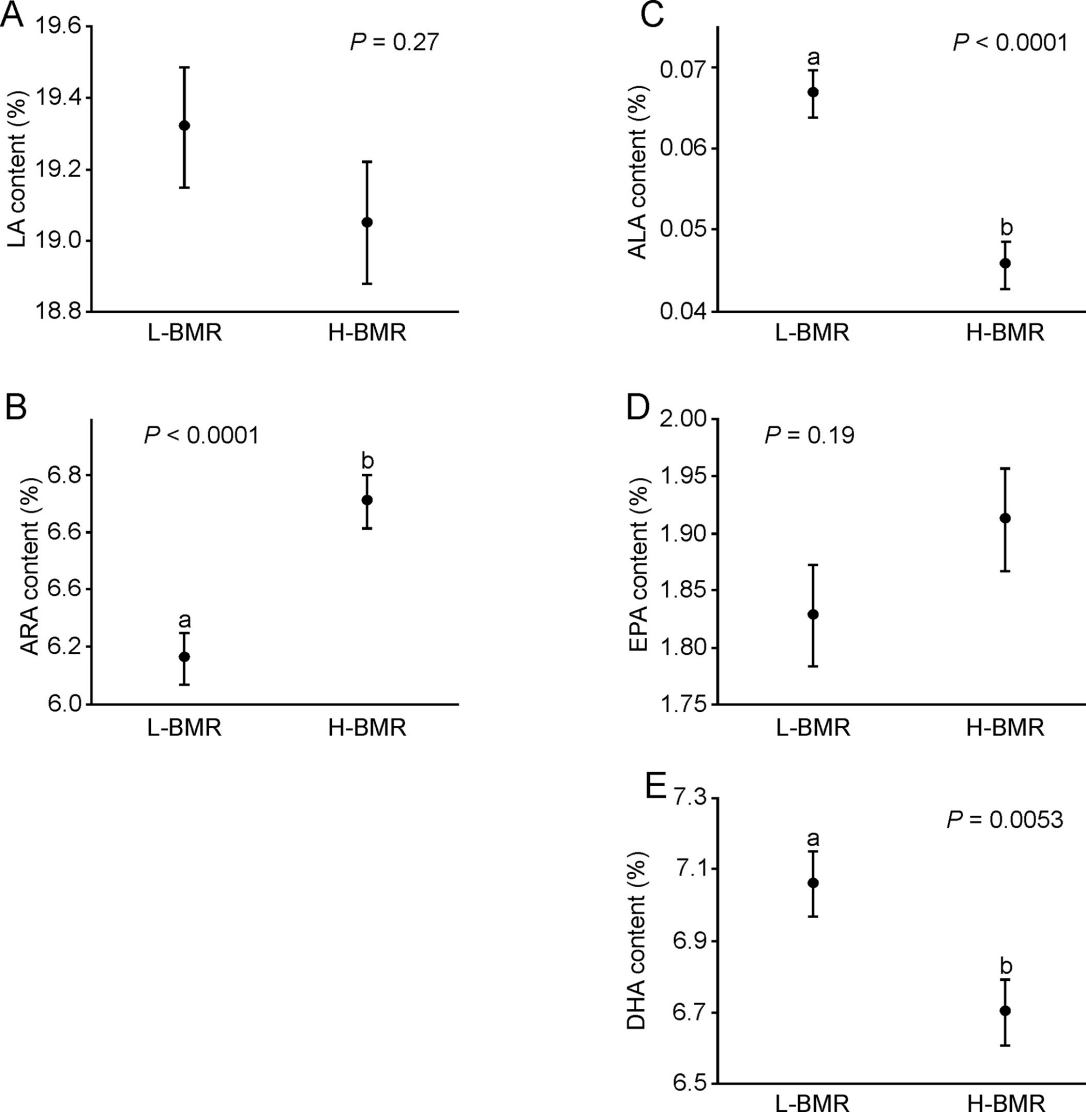
Effect of selection on PUFA abundance in total liver lipids. Data are presented as the means ± s.e.m. Different letters indicate significant differences between lines. Sample size was *n* = 60 for line L-BMR and *n* = 59 for line H-BMR.

**Fig 5:**
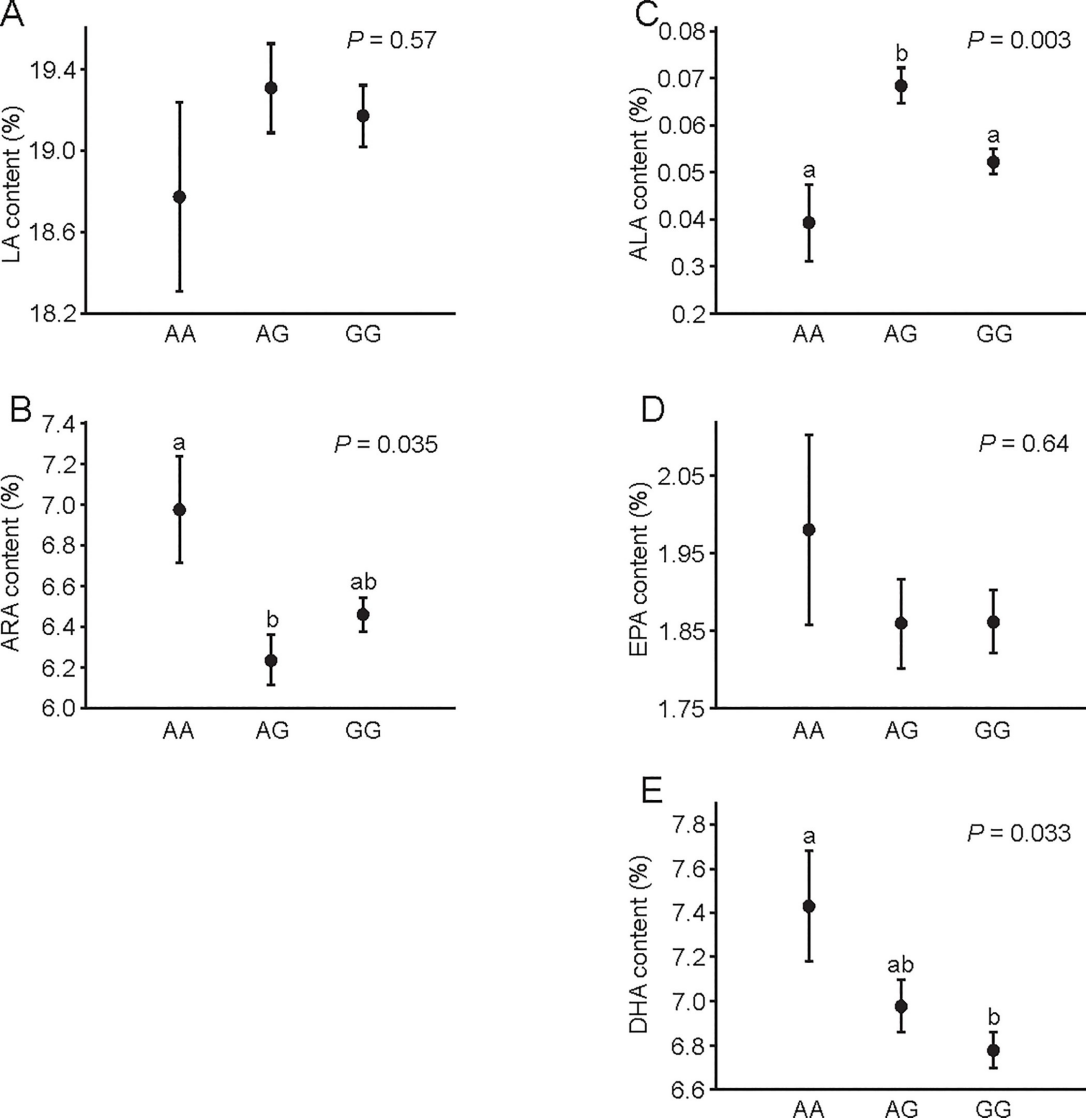
Effect of the *Fads2* genotype on PUFA abundance in total liver lipids. Data are presented as the means ± s.e.m. Different letters indicate significant differences between genotypes. Sample size was *n* = 8 for AA genotype, *n* = 36 for AG genotype, and *n* = 75 for GG genotype.

In F32, mice from the H-BMR line had a higher abundance of ARA but lower ALA and DHA in total liver lipids than did mice from the L-BMR line (Fig 4, Table 2). The abundance of ALA, ARA and DHA in total liver lipids was also affected by the *Fads2* genotype (Fig 5, Table 2). ARA was significantly more abundant in mice with the AA genotype than it was in mice with the AG genotype (*P* = 0.032), whereas GG did not differ from either genotype (*P* > 0.1 for both comparisons). On the other hand, ALA was significantly more abundant in mice with AG than in mice with the AA and GG genotypes (*P* < 0.015 in both cases), whereas mice with the AA and GG genotypes did not differ (*P* = 0.3). Finally, mice with the AA genotype had a higher abundance of DHA than did mice with the GG genotype (*P* = 0.039), whereas mice with the AG genotype showed intermediate values that did not differ from the two homozygous genotypes (*P* > 0.2 for both comparisons).

**Table 2:** Results of ANOVA testing effects of selection and genotype on abundance of PUFAs in total liver lipids. Df is 1,117 for effect of selection and 2,116 for effect of genotype.

	Effect of selection		Effect of genotype		Effect of genotype on standardized values	
	*F*	*P*	*F*	*P*	*F*	*P*
18:2n6 (LA)	1.23	0.27	0.56	0.57	0.58	0.56
18:3n3 (ALA)	26.3	<0.0001	6.13	0.003	5.35	0.006
20:4n6 (ARA)	18.0	<0.0001	3.46	0.035	3.39	0.037
20:5n3 (EPA)	1.78	0.19	0.44	0.64	1.30	0.28
22:6n3 (DHA)	8.08	0.0053	3.51	0.033	2.59	0.079

**Fig 6:**
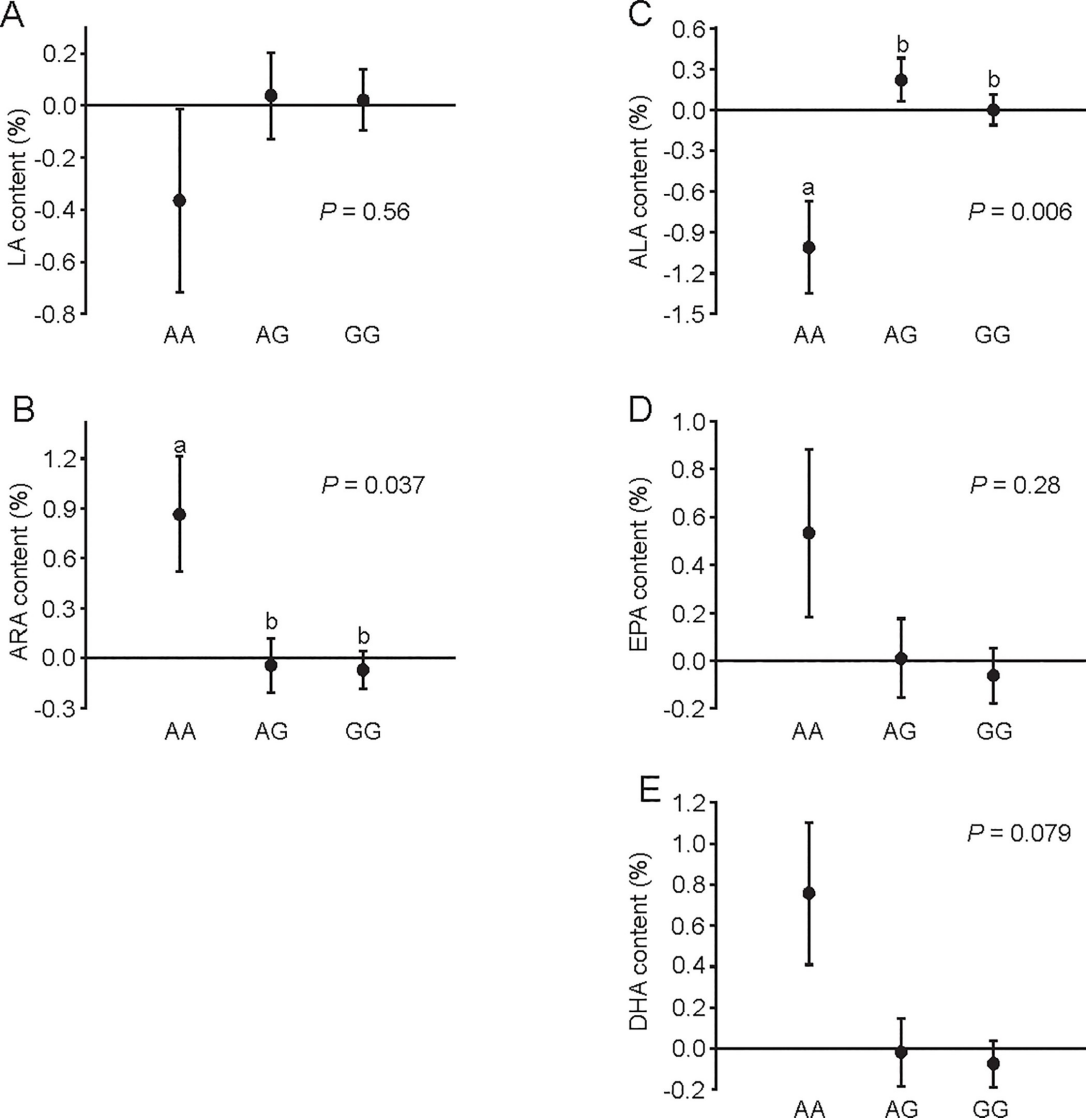
Effect of the *Fads2* genotype on standardized scores of PUFA abundance in total liver lipids. Data are presented as the means ± s.e.m. Different letters indicate significant differences between genotypes. Sample size was *n* = 8 for AA genotype, *n* = 36 for AG genotype, and *n* = 75 for GG genotype.

When calculations were carried out on standardized values, the *Fads2* genotype had significant effects on the standard scores of abundance of ALA and ARA (Fig 6, Table 2). For ALA, there was no difference between genotypes AG and GG (*P* = 0.5), whereas AA was lower than both GG (*P* = 0.015) and AG (*P* = 0.004) were. For ARA, there was no difference between AG and GG (*P* = 0.99), whereas AA was higher than both AG and GG (*P* = 0.048 and *P* = 0.029) were. Mice with the AA genotype tended to have a higher abundance of DHA; however, this effect was not statistically significant (Fig 6, Table 2).

## Discussion

In this study, we investigated the presence of polymorphism in genes controlling n-6 and n-3 PUFA metabolic pathways in mice selected divergently for either high or low BMR. We found one, nonsynonymous single nucleotide polymorphism (G/A) in the *Fads2* gene. Strikingly, allele A occurred significantly more frequently in the L-BMR line than it did in the H-BMR line. Similarly, analysis of genetic distances based upon *F*_ST_ values revealed significant genetic differentiation at the *Fads2* locus between lines L-BMR and H-BMR in both generations studied. However, the studied lines of mice were not replicated because maintaining several independent lines was not feasible due to the workload required by the BMR assays. Therefore, any differences in gene frequency between these two lines may potentially reflect the effect of genetic drift rather than selection [29]. Nevertheless, the genetic differentiation (expressed as *F*_ST_) between lines H-BMR and L-BMR in F22 was significantly greater at the *Fads2* locus than it was at any of the 10 studied noncoding microsatellite loci (Fig 1). However, the average *F*_ST_ for neutral microsatellite loci increased between F22 and F32 (from 0.096 to 0.224), and the *F*_ST_ for *Fads2* in F32 fell within the range of values observed for neutral loci. We conclude that the genetic variation between lines H-BMR and L-BMR developed significantly faster during the course of selection at the *Fads2* locus than at neutral loci, though it was later masked by genetic drift effects. This pattern suggests that genetic polymorphism at the *Fads2* gene is related to variation in BMR, i.e., the direct target of selection.

Among mammalian desaturases, only Δ6-desaturase, encoded by the *Fads2* gene [20], initiates the desaturation-chain elongation cascade by which dietary PUFA precursors linoleic acid (18:2n-6, LA) and α-linolenic acid (18:3n-3, ALA) are transformed to highly unsaturated fatty acids [30]. The observed G/A polymorphism resulted in a protein change located 17 amino acids before the first of three conserved histidine-rich regions and in the first of two transmembrane domains of Δ6-desaturase. This region reportedly binds nonheme iron, which is required for enzymatic activity [31]. Thus, it is likely that the observed SNP may result in two isoforms of Δ6-desaturase with different properties. Desaturases appear to be the rate-limiting enzymes in the synthesis of most PUFAs [7, 32], and genetic polymorphism in *Fads2* can exert significant effects on fatty acyl composition and related physiological parameters [11, 33].

Paradoxically, even though the large difference in the frequency of *Fads2* alleles between the two lines suggests that the polymorphism is related to variation in BMR, it is difficult to directly demonstrate such a link since the effect of the *Fads2* polymorphism cannot be disentangled from the effects of other factors that differentiate both lines. Moreover, single SNPs may have an undetectably small effect on the phenotype in complex polygenic physiological traits such as BMR [34]. Therefore, it is no surprise that we found a significant effect of *Fads2* polymorphism on BMR when we ignored line affiliation (Fig 3A and 3B) but not when we used standardized values of BMR, i.e., focused on intraline variation in BMR (Fig 3C and 3D). If the *Fads2* polymorphism affects BMR through modification of cell membrane properties, one may also expect a significant effect of the *Fads2* genotype on fatty acyl composition of the cell membrane phospholipids. Although we know the fatty acyl composition of liver phospholipids of F22 mice [13], almost all H-BMR mice in this generation possessed the GG genotype (see Results), precluding us from testing the effect of the *Fads2* genotype independently of the line effect. Unfortunately, for F32, we only collected data on fatty acyl composition in total liver lipids. However, phospholipids are the dominant (ca. 80% of total weight) lipid class in the liver of rodents [35]. Moreover, higher ARA and DHA abundance in total liver lipids in the L-BMR line in F32 is similar to the differences observed for phospholipids in F22 [13]. Therefore, we used fatty acyl composition of total liver lipids as a proxy for DHA abundance in liver phospholipids.

The *Fads2* genotype exerted significant effects on the abundance of several PUFAs (Figs 5 and 6, Table 2). The general pattern that emerges from Figs 5 and 6 is that the G allele is dominant and reduces the activity of Δ6-desaturase in both n-3 and n-6 PUFA biosynthesis pathways, since mice possessing this allele usually had more substrates (LA and ALA) and less products (ARA, EPA, DHA) of these pathways. This pattern is particularly evident for the standard scores of PUFA abundance (Fig 6), i.e., for the effect of the *Fads2* polymorphism on within-line variation in PUFA composition. However, the trends we described were significant only for ALA and ARA and near-significant for DHA. We hypothesize that the lack of significance reflects a large standard error in the AA group resulting from a small number of mice with this genotype. Interestingly, studies of genetic polymorphism of the *Fads1*/*Fads2* genes in humans have found that minor alleles usually reduce (reviewed in [11]) and dominant alleles increase [36, 37] the activity of desaturases, whereas our results suggest an opposite pattern for the described SNP here.

Analysis of the *Fads2* genotypes suggests that genotype AA is related to low ALA content and high ARA and DHA content. However, allele A is more common in the L-BMR line, and mice from this line had higher abundance of ALA and DHA and a lower abundance of ARA than did mice from line H-BMR. Thus, the predicted effect of the *Fads2* genotype on PUFA composition was consistent with the observed differences between selected lines for DHA only (compare Fig 4 with Figs 5 and 6). DHA is presumably the main predictor of cell membrane properties and activity of metabolic processes [6, 7]. Therefore, we hypothesize that the described polymorphism in the *Fads2* gene has been affected by selection for BMR because it modulates DHA content (and, consequently, properties of cell membranes and BMR) in accordance with the direction of selection. At the same time, the differences between lines in other biochemical mechanisms (e.g., activity of metabolic pathways that use PUFAs as substrates) may conceal the effect of the *Fads2* polymorphism on the abundance of other PUFAs. Similarly, several SNPs in the *Fads2* gene were found to exert significant effects on the abundance of some PUFAs but not others [33, 37], confirming that the phenotypic effects of *Fads2* alleles may vary among different PUFAs.

To summarize, we described a novel polymorphism in the *Fads2* gene which encodes Δ6-desaturase in mice. The divergent selection for BMR affected allele frequency of the *Fads2* gene, suggesting the presence of a significant link between this polymorphism and variation in BMR. Although we cannot unambiguously identify the molecular mechanism responsible for such a link, we hypothesize that it may reflect modulation of the cell membrane fatty acyl composition (most likely, abundance of the DHA fatty acid). We are not aware of any earlier studies that have found a link between polymorphism in genes responsible for fatty acyl composition and intraspecific variation in BMR. Our results confirm the feasibility of the evolution of BMR through genetic-based changes in fatty acyl composition as proposed by MPTM (although there is an inconsistency between the direction of changes in fatty acyl composition in the studied mice and the predictions of MPTM). They also validated that differences in activity of desaturases may represent an important factor responsible for evolutionary differences in the unsaturation of cell membranes [7, 32].

## Supporting information

S1 Fig Localization of polymorphic sites in the *Fads2* gene of a laboratory mouse (*Mus musculus*). (**A**) Ideogram of chromosome 19 of a laboratory mice (*Mus musculus*); two lines indicate the localization of the gene *Fads2*, which is located between 10,138,654 and 10,175,993 bp in section B of chromosome 19. (**B**) Scheme of the *Fads2* gene; the arrow indicates the direction of gene transcription; exons are represented by shaded rectangles, while introns are represented by dark lines connecting them. (**C**) Exact localization of identified polymorphic sites; codons containing polymorphic sites and their corresponding amino acids are marked by frames. In addition, an nonsynonymous polymorphism is marked in red.
(DOCX)

S1 File Genotype, metabolic, and biochemical data used in the present study. (XLSX)

S1 Table Primers used for PCR amplification and sequencing of the studied genes in mice. (DOCX)

S2 Table Microsatellite loci used in the present study. Two sets (MmI and MmII) of 10 microsatellite loci and their summary statistics calculated for the studied lines of mice.
(DOCX)
