# Supplementary material for: A novel polymorphism in the fatty acid desaturase 2 gene (Fads2): A possible role in the basal metabolic rate
Source: PLoS One. 2019 Feb 28;14(2):e0213138. doi: 10.1371/journal.pone.0213138 (PMC6394981; doi:10.1371/journal.pone.0213138)
Supplement: S1 Fig — (A) Ideogram of chromosome 19 of a laboratory mice (Mus musculus); two lines indicate the localization of the gene Fads2, which is located between 10,138,654 and 10,175,993 bp in section B of chromosome 19. (B) Scheme of the Fads2 gene; the arrow indicates the direction of gene transcription; exons are represented by shaded rectangles, while introns are represented by dark lines connecting them. (C) Exact localization of identified polymorphic sites; codons containing polymorphic sites and their corresponding amino acids are marked by frames. In addition, an nonsynonymous polymorphism is marked in red. (DOCX) [file pone.0213138.s001.docx]

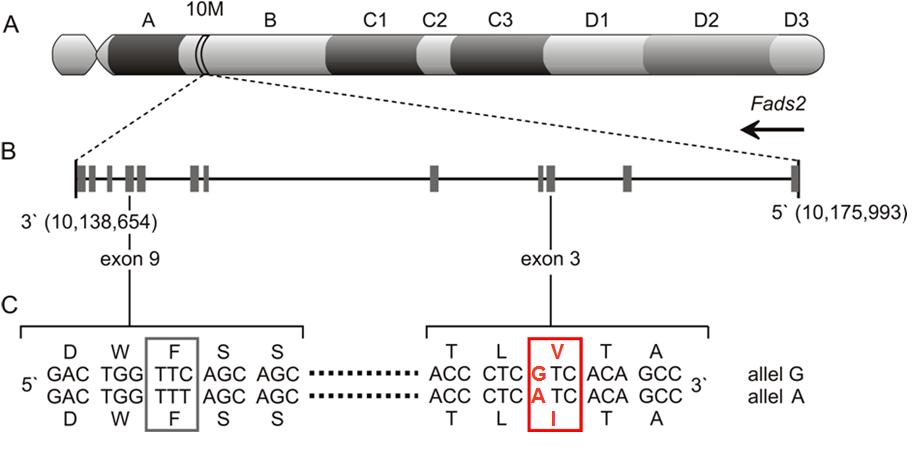


**S1 Fig.** **Localization of** **polymorphic sites in the *Fads2* gene of a laboratory mouse (*Mus musculus*).** (**A**) Ideogram of chromosome 19 of a laboratory mice (*Mus musculus*); two lines indicate the localization of the gene *Fads2*, which is located between 10,138,654 and 10,175,993 bp in section B of chromosome 19. (**B**) Scheme of the *Fads2* gene; the arrow indicates the direction of gene transcription; exons are represented by shaded rectangles, while introns are represented by dark lines connecting them. (**C**) Exact localization of identified polymorphic sites; codons containing polymorphic sites and their corresponding amino acids are marked by frames. In addition, an nonsynonymous polymorphism is marked in red.
