# Supplementary material for: A novel polymorphism in the fatty acid desaturase 2 gene (Fads2): A possible role in the basal metabolic rate
Source: PLoS One. 2019 Feb 28;14(2):e0213138. doi: 10.1371/journal.pone.0213138 (PMC6394981; doi:10.1371/journal.pone.0213138)
Supplement: S1 Table — (DOCX) [file pone.0213138.s003.docx]

**S1 Table.** **Primers used for PCR amplifications and sequencing of the studied genes in mice.**

| **Gene** | **Abbreviation** | **Primers (5’- 3’)** | **Template** | **Product size** | **Product**  **characterization** |
| --- | --- | --- | --- | --- | --- |
| *Fads1* | MmD5D1e  MmD5D5e  MmD5D4e  MmD5D12e | F: TTCGCGAGCCTGGCGTGCGCT  R: ACCTTCCCCAGGGCGAAGAAGA  F: TCGACATGGAATCACCTGCTA  R: TCGAAGGAGTTGCACTGTTCC | cDNA | 818 bp  1145 bp | exons 1-5  exons 4-12 |
| *Fads2* | MmD6D1e  MmD6D12e  MmD6D3e*  MmD6D4e*  MmD6D8e*  MmD6D10e* | F: ATCCGGGTAGACTGGCAGCAT  R: CTCATGAGAACCCTTCAGAACA  F: TCAGATCACCGAGGACTTCA  R: GCCAATGACAAACTTGTGGAC  F: GGCTTGGGCCATCAGCTACTAT  R: TGGTGCTCAATCTGGAAAT | cDNA  gDNA  gDNA | 1357 bp  306 bp  1006 bp | exons 1-12  exons 3, 4  intron 3  exons 8, 9, 10  introns 8, 9 |
| *Elovl-2* | Mm2El1e  Mm2El8e | F: CTACCCTGGACAGCGCAT  R: CGGCATGGCTCCAGTTAGTA | cDNA | 1116 bp | exons 1-8 |
| *Elovl-5* | Mm5El1e  Mm5El7e | F: TTGCATCGCGGCTGCAGCTT  R: TCACACTGCAGATAGTAGGGCC | cDNA | 1131 bp | exons 1-7 |

* - primers used for amplification of sequences with polymorphic sites
