# Supplementary material for: A novel polymorphism in the fatty acid desaturase 2 gene (Fads2): A possible role in the basal metabolic rate
Source: PLoS One. 2019 Feb 28;14(2):e0213138. doi: 10.1371/journal.pone.0213138 (PMC6394981; doi:10.1371/journal.pone.0213138)
Supplement: S2 Table — Two sets (MmI and MmII) of 10 microsatellite loci and their summary statistics calculated for the studied lines of mice. (DOCX) [file pone.0213138.s004.docx]

**S2 Table.** **Microsatellite loci used in the present study.** Two sets (MmI i MmII) of 10 microsatellite loci and their summary statistics calculated for the studied lines of mice.

| **Set** | **Locus** | **Primers (5’- 3’)** | **Dye** | **Size range** |
| --- | --- | --- | --- | --- |
| **MmI** | D1Mit322* | F: CAAATTTACACCCATGTTGTGG | VIC | 324-332 |
|  |  | R: TCAATGGAGGGGAAGATCAG |  |  |
|  | D5Mit95* | F: TGTTCTTGTCCATGTCTGATCC | VIC | 118-134 |
|  |  | R: AACCAAAGCATGAAACAGCC |  |  |
|  | D12Mit4* | F: ACATCCCCAGCTCTTGTTTG | FAM | 184-207 |
|  |  | R: AAACCAAACCAAAGAAGCTTAGG |  |  |
|  | D17Mit51* | F: TCTGCCCTGTAACAGGAGCT | PET | 141-157 |
|  |  | R: CTTCTGGAATCAGAGGATCCC |  |  |
|  |  |  |  |  |
| **MmII** | D6Mit138* | F: GCTCTTATTAATGAAGAAGAAGAAGG | VIC | 126-138 |
|  |  | R: CAAAGAAAGCATTTCAAGACTGC |  |  |
|  | D10Mit20* | F: CACCCTCACACAGATATGCG | NED | 218-238 |
|  |  | R: GCATTGGGAAGTCCATGAGT |  |  |
|  | D15Mit16* | F: AGACTCAGAGGGCAAAATAAAGC | PET | 119-135 |
|  |  | R: TCGGCTTTTGTCTGTCTGTC |  |  |
|  | MUSMCKA(D7)** | F: CCAGACCATCTGATCCAGATC | FAM | 123-139 |
|  |  | R: GGAGGTTGCAGTGAATTCAAG |  |  |
|  | MMCY03(D9)** | F: AGTTTTAGGCTAGTATAGGTT | FAM | 188-200 |
|  |  | R: ACTGGAACCTTAGAGCATGAG |  |  |
|  | MMGFAPD(D11)** | F: GTACTAAAACGTCTACAAGTGG | NED | 96-100 |
|  |  | R: GCGGATATATATGCAGCAGAG |  |  |

References:

* www.informatics.jax.org

** C.M. Hearne, M.A. McAleer, J.M. Love, et al., Additional microsatellite markers for mouse genome mapping, Mamm. Genome. 1 (1991) 273–282.
